# Worldwide survey on the transcervical approach for minimally invasive treatment of esophageal cancer: results of questionnaire of the international collaborative group on transCervical Minimally Invasive Esophagectomy

**DOI:** 10.1093/dote/doaf035

**Published:** 2025-05-18

**Authors:** Federica Riccio, Bastiaan R Klarenbeek, Hitoshi Fujiwara, Yasuyuki Seto, Miguel A Cuesta, the International CMIE Collaborative Group, Hiroyuki Daiko, Peter P Grimminger, Yang Hu, Qingdong Cao, Takashi Mitsui, Kazuhiko Mori, Atsushi Shiozaki, Koichi Ogawa, Yaxing Shen, Tomotaka Shibata, Yasuhiro Shirakawa, Yutaka Tokairin

**Affiliations:** Department of Surgery, Radboud University Medical Center, Nijmegen, the Netherlands; Department of Surgery, Oncology and Gastroenterology, 1st Surgical Clinic, University of Padova, Padova, Italy; Department of Surgery, Radboud University Medical Center, Nijmegen, the Netherlands; Division of Digestive Surgery, Department of Surgery, Kyoto Prefectural University of Medicine, Kyoto, Japan; Department of Gastrointestinal Surgery, Graduate School of Medicine, Tokyo, Japan; Department of Surgery, Amsterdam University Medical Centers, Amsterdam, the Netherlands; Division of Esophageal Surgery, National Cancer Center Hospital, Tokyo, Japan; Department of General, Visceral and Transplant Surgery, University Medical Centre Mainz, Mainz, Germany; Department of Thoracic Surgery, West China Hospital, Sichuan University, Chengdu, China; Department of Thoracic Surgery, The Fifth Affiliated Hospital of Sun Yat-sen University, Zhuhai, China; Department of Surgery, Dokkyo Medical University Saitama Medical Center, Saitama, Japan; Department of Gastrointestinal Surgery, Mitsui Memorial Hospital, Tokyo, Japan; Division of Digestive Surgery, Department of Surgery, Kyoto Prefectural University of Medicine, Kyoto, Japan; Department of Gastrointestinal and Hepato-Biliary-Pancreatic Surgery, Faculty of Medicine, University of Tsukuba, 1-1-1, Tennodai, Tsukuba, Ibaraki 305-8575, Japan; Department of Thoracic Surgery, Zhongshan Hospital, Fudan University, Shanghai, China; Department of Gastroenterological Surgery, Faculty of Medicine, Oita University, Yufu, Japan; Department of Surgery, Hiroshima City Hiroshima Citizens Hospital, Hiroshima, Japan; Department of Surgery, Toshima Hospital Tokyo Metropolitan Health and Hospitals Corporation, Tokyo, 173-0015, Japan

**Keywords:** esophageal cancer, esophagectomy, mediastinoscopy, minimally invasive, transcervical

## Abstract

Minimally invasive esophagectomy has emerged over open surgery, showing important advantages on the short time and comparable oncological outcomes, though concerns persist about pulmonary complications. The avoidance of the thoracic route has shown lower pulmonary complication rates compared to traditional approaches. Since 2015, a novel technique was introduced combining single port transcervical and transhiatal laparoscopic mediastinal dissection for esophageal resection. The procedure is nowadays expanding, following the IDEAL framework stage 2b an international collaborative group has been created to register, standardize the procedure, selection of patients, and assess the outcome, highlighting the need for ongoing discussion and research. A broad questionnaire was sent to all the groups worldwide known to us performing this procedure. Results of this questionnaire are the goal of this manuscript, aiming to present the procedure, experiences, problems, and lessons learned with this procedure. The thirteen respondents currently practicing this technique were categorized based on case volume and experience. Results show varied surgical indications, with higher-volume centers displaying less selectivity. Slightly different surgical approaches were reported, with the left transcervical and abdominal transhiatal dissection being the basis techniques. Surgeons reported high rates of R0 resection and lymph node harvested, low rates of pulmonary complications and anastomotic leaks, but high rates of recurrent laryngeal nerve injuries. The collaborative group aims to align techniques, share experiences, and standardize procedures. Despite concerns about recurrent laryngeal nerve injuries, not different from other total mediastinal lymphadenectomy techniques, the procedure shows promise in reducing pulmonary complications, marking a crucial step toward global acceptance. Future efforts will focus on standardization, comparative studies, and training protocols.

## INTRODUCTION

Esophageal cancer is one of the leading causes of cancer-related mortality worldwide.^1^ The treatment algorithm for locally advanced esophageal cancer mainly consists of the association of neoadjuvant chemoradiotherapy or perioperative chemotherapy with surgical resection.^2^^,^^3^

Esophagectomy with two or three field lymphadenectomy is nowadays the standard of care but is still related to a high rate of complications that can impact long-term survival after surgery.^4^ Transthoracic minimally invasive esophagectomy,^5^ is associated with fewer overall and pulmonary postoperative complications, a shorter hospital stay, and higher Quality of Life outcomes, while maintaining similar oncological quality^6^^,^^7^ and comparable survival to open surgery.^8^ Despite these positive outcomes, the rate of pulmonary complications remains high (up to 28.3%).^9^

In 2015 a new technique for esophageal resection and complete mediastinal lymph node dissection was described by Fujiwara et al.,^10–12^ combining a single-port transcervical and transhiatal laparoscopic mediastinal dissection and reconstruction by a gastric conduit with cervical anastomosis. Currently, the procedure is expanding in different centers around the world, mainly Japan and China, but recently also in the Netherlands and Germany. Although these centers use a similar approach, there are differences in selection of patients for this procedure, operative technique employed, devices used, and results.

During the 19th ISDE World Congress in Toronto, an international collaborative group has been created to know each other, to register the patients, and to try to solve the points above-mentioned. Moreover, announcing the procedure, publication of results, and training purposes are the goals of the group.

According to the existing data in the literature, the results are promising, showing very low pulmonary complication rates (9%) compared to the transthoracic approach and similar results for overall complications (37.8%) and oncological safety (mean number of harvested lymph nodes 23.38 and rate of R1 resection of 3.7%).^13^

Introducing and implementing a new surgical technique involves a steep learning curve for surgeons. The IDEAL framework,^14^ comprising five steps, offers a useful tool in this process to mitigate errors correlated with development and to establish the best practices for patient treatment. A few centers have pioneered transCervical Minimally Invasive Esophagectomy (CMIE) and reported their initial results as part of stage 2a development according to the IDEAL framework. To position this technique amongst other minimally invasive procedures for esophageal cancer surgery and prepare for comparative and randomized studies (stage 2b), it is important for the international collaborative group to align their technique with each other and find consensus on important outcomes and definitions.

The goal of this overview paper is to describe the status and position of CMIE and present the current indications, operative technique, and results to the world of Upper GI surgeons.

## MATERIAL AND METHODS

A cross-sectional survey was sent to evaluate the surgeon’s preferences and outcomes while performing the CMIE procedure. The English survey (supplementary materials) was elaborated by one of the investigators and reviewed by three experts in minimally invasive esophageal surgery. The questionnaire consisted of six sections: (i) general information and learning process; (ii) indication and selection of patients; (iii) surgical technique; (iv) post-operative management; (v) complications; and (vi) surgical outcomes. Invitations were initially sent to nineteen surgeons from sixteen different centers (list of the invited centers available in Supplementary Materials), who, according to our information, are currently performing worldwide minimally invasive esophagectomy with a transcervical and transhiatal approach. After a second sending to the non-responders, six surgeons were excluded. On 28 November 2023, an online meeting of the group, consisting surgeons of thirteen centers, took place, and the results of the survey were discussed. Moreover, during this first online meeting, the surgeons had the chance to present their technique to the other members of the group with a short video presentation.

### Statistical analysis

All data were analyzed anonymously. When appropriate, statistical analysis was performed using IBM SPSS statistics (version 29.0 for Windows). The referred mean operative time, mean blood loss, rate of anastomotic leak, rate of pulmonary complication, and rate of recurrent laryngeal nerve (RLN) injuries were compared for surgeon’s case volume (number of cases/year) and experience (total number of cases).

### Surgical technique

The procedure is carried out with the patient in the supine position, utilizing bilateral lung ventilation. A 4 cm left collar incision is made, 1 cm over the manubrium and clavicle, crossing 1–2 cm from the midline and 1–2 cm from the medial edge of the left sternocleidomastoid muscle. Through this incision, the cervical esophagus is dissected, and para-esophageal cervical lymph nodes are harvested, while identifying the left RLN. Subsequently, a single-port device is placed, and pneumomediastinum (8–10 mmHg pressure) inducted.

The dissection proceeds on the left side along the left RLN, progressing toward the left main bronchus. Ventrally, the esophagus is dissected from the membranous trachea until reaching the subcarinal space for lymphadenectomy. On the right side, lymph nodes along the right RLN are dissected, followed by dissection along the right pleura. The dissection is completed dorsally.

A laparoscopic gastric mobilization with abdominal D1+ lymphadenectomy is performed to create a gastric conduit, concurrently with transhiatal dissection of the lower esophagus to connect the two dissection fields (transcervical and transhiatal). The gastric conduit is pulled up via the posterior mediastinum or retrosternal. Reconstruction is carried out in the neck through an esophagogastric anastomosis.

There might be variations to this technique among the individual centers.

## RESULTS

### Demographics

Out of the nineteen invited surgeons, thirteen surgeons (68.4%), representing thirteen different centers, responded to the survey. The list of participating centers is provided in the supplementary materials. The respondents were from Japan (*n* = 9), China (*n* = 2), Germany (*n* = 1), and the Netherlands (*n* = 1).

Among the responders, two surgeons had less than five years of experience performing CMIE, nine surgeons had between 5 and 10 years of experience, and two surgeons had over 10 years of experience. Based on their volume (cases/year), surgeons were categorized into four groups: less than 10 cases (*n* = 3), between 10 and 19 cases (*n* = 3), between 20 and 29 cases (*n* = 4), and more than 30 cases (*n* = 3). Additionally, they were divided into three different groups according to their global experience: less than 100 cases (*n* = 5), between 100 and 199 cases (*n* = 5), and more than 200 cases (*n* = 3). The distribution of the various groups based on their countries is presented in Table 1.

**Table 1:** Volume and experience of the responders

***n* (%)**		**Country**
Volume (cases/year)		
<10	3 (23.1)	Germany (1), Japan (2)
10–19	3 (23.1)	Japan (3)
20–29	4 (30.7)	China (1), Japan (2), The Netherlands (1)
≥30	3 (23.1)	China (1), Japan (2)
Experience (total number of cases)		
<100	5 (38.5)	Germany (1), Japan (3), The Netherlands (1)
100–199	5 (38.5)	China (1), Japan (4)
≥200	3 (23.0)	China (1), Japan (2)

### Learning process

Twelve surgeons from twelve different centers attended as observers to learn CMIE at two centers (three at the University of Tokyo, eight at Kyoto Prefectural University of Medicine Hospital, and one in both centers). Before starting the procedure on patients, six surgeons from six different centers received further cadaver training, and only one surgeon used only the cadaver simulation for training.

### Indications

**Fig. 1:**
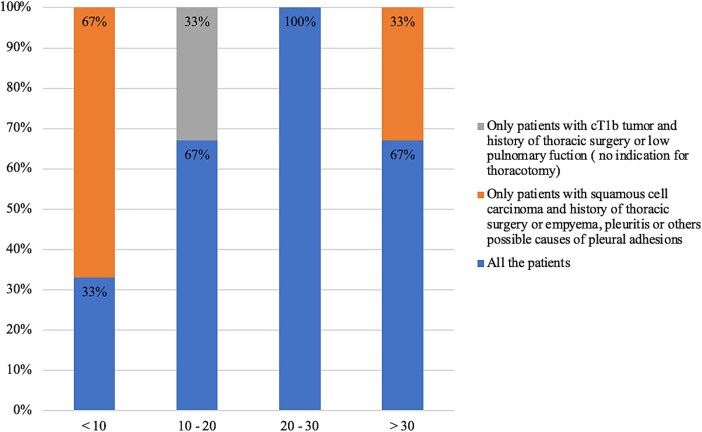
Indications for CMIE according to volume.

Indications for CMIE are depicted in Table 2. Surgeons from high-volume centers tend to be less selective in choosing eligible patients for CMIE (Fig. 1). General contraindications include large tumors near the carina, bulky primary lesions, or T4 tumors.

**Table 2:** Indications for CMIE according to country

**Indications**	***n* (%)**	**Country**
All patients	9 (69.2)	China (1), Japan (7), The Netherlands (1)
Only in patients with squamous cell carcinoma and history of thoracic surgery or of disease that can cause pleural adhesion	3 (23.1)	China (1), Germany (1), Japan (1)
Only patient with cT1b tumors and history of thoracic surgery or low pulmonary function	1 (7.7)	Japan (1)

Regarding upfront or neoadjuvant therapy, CMIE is utilized in patients treated with neoadjuvant chemo-radiation therapy in seven centers (53.8%), while in the other six centers (46.2%) it is only employed as an upfront treatment or for patients receiving neoadjuvant chemotherapy.

### Surgical approach and intraoperative data

Data are shown in Table 3. Different approaches are used: single-port left transcervical mediastinoscopy and transhiatal laparoscopic or robotic dissection in nine centers; transcervical and transhiatal fully robotic in one; bilateral single-port transcervical mediastinoscopy and laparoscopic or robotic abdominal dissection in two; and left single-port mediastinoscopy plus a right cervical auxiliary trocar and laparoscopic transhiatal dissection in one.

**Table 3:** Surgical approach and intraoperative data according to the country. IONM (intraoperative neuromonitoring)

**Intraoperative data**	***n* (%)**	**Country**
Surgical approach		
Single port left transcervical mediastinoscopy and laparoscopic or robotic abdominal dissection	9 (69.2)	China (1), Japan (7), The Netherlands (1)
Fully robotic	1 (7.7)	Germany (1)
Bilateral single port transcervical mediastinoscopy and laparoscopic or robotic abdominal dissection	2 (15.4)	Japan (2)
Left primary single port mediastinoscopy plus a right cervical auxiliary trocar and laparoscopic abdominal dissection	1 (7.7)	China (1)
Order of phases		
Transcervical and then trans hiatal	5 (38.4)	Japan (4), The Netherlands (1)
Trans hiatal and then transcervical	1 (7.7)	Germany (1)
Simultaneously	4 (30.8)	China (1), Japan (3)
Variable	3 (23.1)	China (1), Japan (2)
Pneumomediastinum pressure		
Fixed on 8 mmHg	2 (15.4)	Germany (1), The Netherlands (1)
Fixed on 10 mmHg	2 (15.4)	China (1), Japan (1)
Progressive pressure from 6 to 10 mmHg	2 (15.4)	Japan (2)
Variable pressure	6 (46.1)	Japan (6)
Not used	1 (7.7)	China (1)
Scope		
Flexible tip	6 (46.2)	Japan (6)
Conventional scope	5 (38.4)	Germany (1), Japan (3), The Netherlands (1)
Both flexible tip and conventional	1 (7.7)	Japan (1)
Gastroscope	1 (7.7)	China (1)
Energy device		
Bipolar vessel sealing device	12 (92.3)	Japan (9), China (1), Germany (1), The Netherlands (1)
Endoscopic hybrid knife	1 (7.7)	China (1)
IONM		
Yes	8 (61.5)	Japan (6), Germany (1), The Netherlands (1)
Not	5 (38.5)	China (2), Japan (3)
Piloromiotomy/pyloroplasty		
Yes	3 (23.1)	China (1), Japan (2)
Not	10 (76.)	China (1), Germany (1), Japan (7), The Netherlands (1)
Anastomosis method		
Handsewn	4 (30.8)	China (1), Japan (2), The Netherlands (1)
Circular stapled	5 (38.5)	China (1), Japan (4)
Linear stapled	3 (23.0)	Japan (3)
Both circular or linear stapled	1 (7.7)	Germany (1)
Route for reconstruction		
Orthotopic	7 (53.8)	China (1), Japan (5), The Netherlands (1)
Retrosternal	5 (38.5)	China (1), Japan (4)
Both	1 (7.7)	Germany (1)

IONM, intraoperative neuromonitoring.

In eight centers (61.5%) intraoperative neuromonitoring (IONM) is employed (two intermittent, four continuous, two both types) to identify and prevent damage to the left RLN. Additionally, nine surgeons tape the left RLN for gentle retraction and to avoid any injury.

The mean reported operative time was about 382 ± 117 minutes, and the mean reported blood loss was 100 ± 71 mL. No statistical differences were observed when analyzing for volume (Table 4) or experience (Table 5).

**Table 4:** Surgical outcomes and complications compared for volume

**Outcome (mean ± SD)**	**<10 cases (*n* = 3)**	**10–19 cases (*n* = 3)**	**20–29 cases (*n* = 4)**	**≥30 (*n* = 3)**	***P***
Surgical outcomes					
Operative time (minute)	380.0 ± 192.9	390.0 ± 108.2	422.0 ± 114.3	323.3 ± 80.2	0.798
Blood loss (mL)	75.0 ± 95.3	98.3 ± 47.5	110.8 ± 79.3	113.3 ± 90.2	0.927
Complications					
Anastomotic leak	8.3 ± 5.8	9.3 ± 1.2	7.3 ± 4.0	10.0.3 ± 4.0	0.828
Pulmonary complication	10.0 ± 5.0	9.7 ± 5.7	11.6 ± 7.7	8.7 ± 2.3	0.922
Recurrent laryngeal nerve injury	25.0 ± 13.3	40.0 ± 26.5	27.8 ± 18.6	15.7 ± 12.0	0.486

**Table 5:** Surgical outcomes and complication compared for experience

**Outcome (mean ± SD)**	**<100 cases (*n* = 5)**	**100–200 cases (*n* = 5)**	**>200 cases (*n* = 3)**	***P***
Surgical outcomes				
Operative time (minute)	396.0 ± 138.1	397.4 ± 135.1	333.7 ± 64.6	0.754
Blood loss (mL)	75.0 ± 69.6	112.0 ± 78.0	122.7 ± 76.0	0.628
Complications				
Anastomotic leak	8.4 ± 4.2	8.4 ± 3.2	9.3 ± 5.0	0.942
Pulmonary complication	8.0 ± 4.5	10.7 ± 3.8	12.7 ± 8.3	0.482
Recurrent laryngeal nerve injury	39.0 ± 22.5	21.0 ± 12.6	17.7 ± 9.3	0.179

Reasons for conversion to right thoracoscopy or thoracotomy are deemed necessary citing cases such as mediastinal bleeding, tracheobronchial injury, hemodynamic instability, tumor infiltration, and other problems.

### Oncological radicality

The rate of R0 resection is reported to be higher than 90% by nine surgeons (69.2%), between 80 and 90% by three surgeons (23.1%), and between 70 and 80% by one surgeon (7.7%).

On average, between 15 and 25 lymph nodes were obtained by two surgeons, 25 to 35 lymph nodes by four, and more than 35 lymph nodes by seven surgeons.

### Postoperative data

The mean length of stay on the intensive care unit was 1.6 ± 1.3 days, and the mean length of hospital stay was 14.6 ± 5.4 days. Oral intake was initiated on average after 5.5 ± 1.9 days postoperatively.

**Fig. 2:**
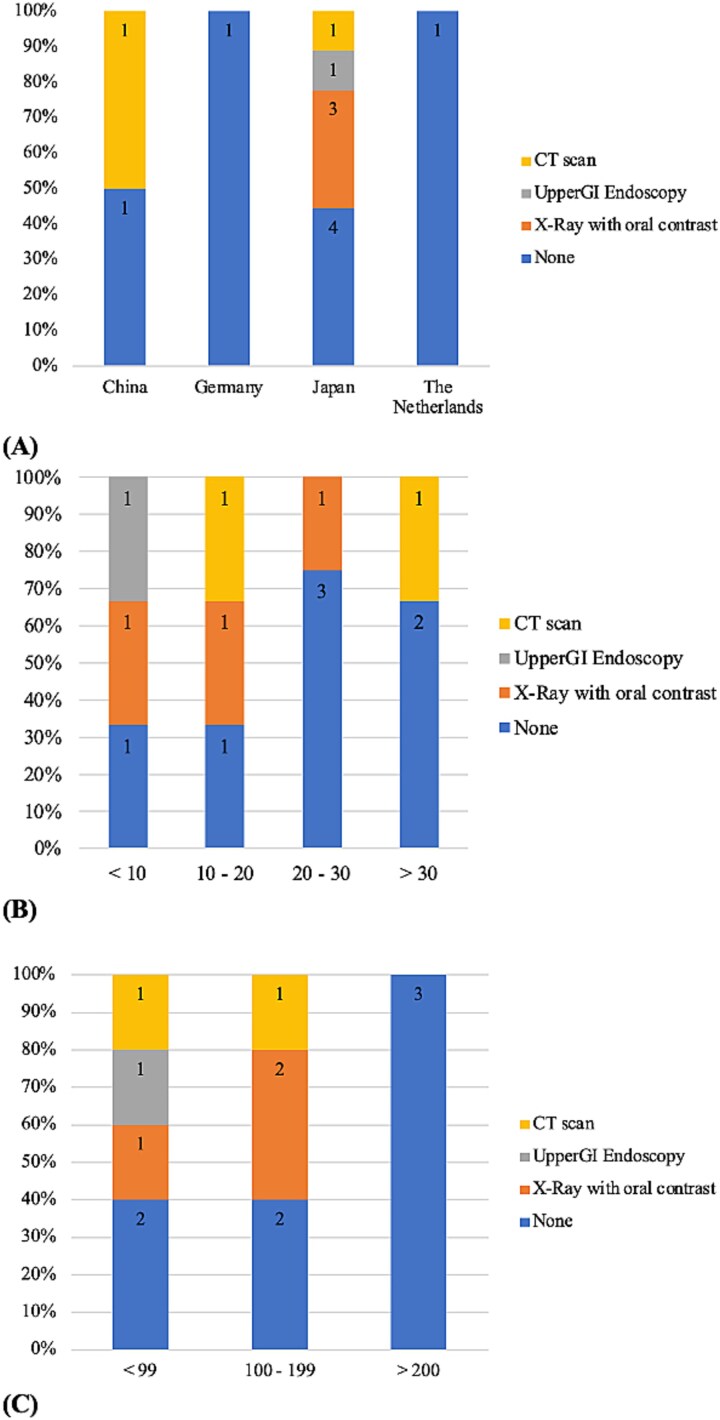
Diagnostic modalities used prior to allow the reprisal of oral intake (A) according to country, (B) according to volume, and (C) according to surgical experience.

Postoperatively, anastomosis checks prior to initiating oral intake were performed in six centers using different tests: oral contrast x-ray (*n* = 3), upper GI endoscopy (*n* = 1), or CT scan (*n* = 2). Figure 2 depicts the use of these diagnostic modalities based on country, volume, and surgical experience. As surgical experience improves and centers handle higher volumes, there is a propensity to not use any diagnostic modalities prior to restarting oral intake.

The mean rates of anastomotic leak, pulmonary complications, and RLN palsies were 8.6 ± 3.7, 10.1 ± 5.2, and 27.2 ± 18.2, respectively. No statistical differences were observed when analyzing for volume (Table 4) or experience (Table 5). However, we observed a decrease in the rate of RLN injuries when the experience increased.

Concerning RLN palsies, 77% were classified as temporarily requiring no therapy (62% type IA and 15% IB according to the Esophagectomy Complications Consensus Group (ECCG) classification^15^), while 23% necessitated an elective surgical procedure (only type IIA according to the ECCG classification^15^).

## DISCUSSION

The combination of the minimally invasive transcervical approach using a cervical platform with the laparoscopic transhiatal procedure is gradually gaining global recognition. This is attributed to the decrease in pulmonary complications. However, its application remains predominantly confined to a limited number of centers, primarily in Eastern countries.

Sixteen groups performing this transcervical approach in various ways have organized into an international collaborative group on CMIE. This group allows for the collective identification and analysis of errors, enabling the development of strategies to prevent and address them. Through discussions, consensus-building, and the exchange of knowledge, surgeons can collectively define optimal approaches, refine surgical protocols, and enhance overall procedural efficiency. The establishment of this CMIE collaborative group now appears essential to facilitate widespread knowledge and eventual adoption.

Standardizing a new surgical technique and outcomes is crucial for ensuring consistency and reproducibility across different surgical settings. This standardization not only enhances patient safety but also sets the stage for spreading the technique to other centers, future research, and comparative studies.

Comparative research is essential for evaluating the effectiveness and safety of the new surgical technique in relation to existing approaches. The collaborative group serves as a foundation for structured, multicenter studies following stage 2b of the IDEAL framework.^14^

We learned from the results of this questionnaire that this procedure is more used in Eastern countries. Most surgeons are performing it liberally for patients with thoracic esophageal cancer, while a small minority of four centers is still restricting its indication to patients for whom a transthoracic approach is not recommended.

The most common practice is to perform CMIE via single-port left transcervical mediastinoscopy and laparoscopic transhiatal dissection, laparoscopic or robotic, using the pneumomediastinum settled on a maximal pressure of 10 mmHg.

The 69.2% of the surgeons had a R0 resection rate higher than 90%. Moreover, all surgeons harvested more than 15 mediastinal lymph nodes.

The greatest difference from the classical transthoracic approach is the different surgical anatomy. This procedure put the surgeons in a challenging, narrow space with a significant limit in terms of amplitude of movements and identification of the correct plane for the dissection. The dissection planes diverge significantly from those in the traditional transthoracic technique. That justifies the choice of most of the surgeons to have an observership and training with fresh frozen cadavers before starting to perform the procedure on real patients. The two centers, with an experience of over 400 cases, were frequently visited by surgeons from the other centers to observe the procedure. Thanks to this collaboration, surgeons can be proctored by experts to expedite the learning curve and receive constructive feedback, minimizing risks associated with the introduction of the CMIE procedure.

The indications reported are quite similar between the centers, with few exceptions. However, we observed that in the centers with a higher volume, there is less patient selection. It exists a relation between the volume and experience of the surgeons with this technique and the selection of patients.

Slightly different approaches to the mediastinum are described by the surgeons. Most surgeons (76.9%) consider the left unilateral transcervical approach sufficient to achieve proper mediastinal dissection and adequate lymphadenectomy on both sides. The dissection of station 106recR from the left side may be considered challenging.^16^ That’s why three surgeons use a bilateral approach. These three centers are all located in Asia, where, due to the higher incidence of upper/middle esophageal squamous cell carcinoma, ensuring complete mediastinal lymphadenectomy is mandatory.

Twelve surgeons use the pneumomediastinum with 8–10 mm Hg for dissection. Only one surgeon uses a different technique based on esophageal mobilization using injection of saline solution and intermittent CO_2_ insufflation through an endoscope.^17^

With this questionnaire, we analyzed three principal post-operative complications: (i) pulmonary, (ii) anastomotic leak, and (iii) RLN injury. In their first series published in literature, Fujiwara et al.^18^ reported rates of pulmonary complications, anastomotic leaks, and RLN palsies of 6.7%, 15.0% and 33.3%. These results are comparable with the findings of this questionnaire: anastomotic leakage (8.6 ± 3.7), pulmonary complications (10.1 ± 5.2), and RLN palsies (27.2 ± 18.2).

The results concerning pulmonary complications are better than any other approach for esophagectomy.^9^

One of the major concerns of this procedure, but also of all procedures involved with complete lymphadenectomy along the RLN, is the rate of RLN injuries. The percentage of RLN palsies in this survey was 27.2%, in over 90% of cases unilateral and temporarily.

The identification of the RLN in the neck, dissection, and gentle manipulation of the nerve, and the devascularization associated with the dissection of the nerve for an optimal lymphadenectomy, could be the influencing factors on this outcome. Importantly, surgeons performing CMIE are studying how to reduce these numbers while conducting a complete supracarinal lymphadenectomy. Nowadays, 84.6% of the surgeons are using tools to identify and preserve the RLNs, employing techniques such as taping of the nerves, IONM, or both.

The use of IONM during esophagectomies is already reported as effective in decreasing the rate of RLN injuries.^19^ When performing minimally invasive transthoracic esophagectomy with three field lymphadenectomies, the use of IONM is associated not only with a reduced rate of injuries of the nerve but also with a more extensive lymphadenectomy (*P* = 0.04).^20^ Komatsu et al.^21^ compared two groups of CMIE patients, without and with continuous IONM and found that it can decrease the rate of RLN palsy from 31.2% to 4.0%. From the results of this survey, it has emerged that three surgeons do not provide this technology, and two others don’t think it will be an advantage for their patients, but we believe that IONM should be encouraged to prevent the rate of RLN injuries.

Additionally, 67% of the surgeons perform a systematic laryngoscopy after this procedure, even in the absence of symptoms. When analyzing the mean rate of RLN injuries by the group who performs the laryngoscopy only in case of symptoms, the rate is lowered to 14.8 ± 11.1. Timing of laryngoscopy evaluation is also important, as the vocal cord palsy is recovered day by day if the RLN injury is mild. We think that perhaps a new definition of this complication is needed when talking of this surgical technique, as a temporary unilateral injury with no or slight symptoms, sequelae, and effects on the outcomes could be common and acceptable.

Moreover, a standard and less traumatic dissection of the RLN with the same radical lymphadenectomy will be the topic of the next meeting of the group.

Some limitations apply to this study. It is a global survey based on retrospective data reported by the interviewees. The number of respondents is relatively low, influenced by the novelty of the technique, which, to the best of our knowledge, is employed in only sixteen centers worldwide. The results are inevitably affected by heterogeneity of the populations involved—varying indications, diverse neoadjuvant treatments, and different postoperative protocols adopted across centers—as well as by the surgeons’ varying levels of experience. Surgical and postoperative outcomes are derived from rates reported by the surgeons, potentially leading to over- or underestimation of the actual data.

Regarding some questions in the survey, they were generally asked, such as indications to convert, but not about the percentage or the reason for conversion. The data concerning the R0 rates are not very clear; however, we requested it as a percentage rather than specific numbers.

Future perspectives of this collaborative group will be explored in upcoming meetings focusing on specific topics such as indications, determining the best surgical approach (unilateral vs. bilateral transcervical, simultaneous versus non-simultaneous mediastinal dissection, conventional MIE, or robotic assisted), and establishing precise definitions for the complications, with the goal of achieving standardization. Once a standard protocol is established, international data collection can start to conduct prospective multicenter exploratory cohort studies and multicenter randomized controlled trials. Additionally, discussion will include the creation of a training protocol to mentor other surgeons in adopting this technique and exploring the limited current role of robotics in this procedure.

In summary, the establishment of the CMIE collaborative group marks a significant stride toward the global adoption of transcervical and transhiatal esophagectomy. Through shared experiences, consensus-building, and standardization efforts, this technique holds the potential to improve patient outcomes and redefine the landscape of esophageal cancer surgery.

## Supplementary Material

Supplementary_material_doaf035(1)
