# Supplementary material for: Worldwide survey on the transcervical approach for minimally invasive treatment of esophageal cancer: results of questionnaire of the international collaborative group on transCervical Minimally Invasive Esophagectomy
Source: Dis Esophagus. 2025 May 18;38(3):doaf035. doi: 10.1093/dote/doaf035 (PMC12085945; doi:10.1093/dote/doaf035)
Supplement: Supplementary_material_doaf035(1) [file supplementary_material_doaf035(1).docx]

Supplement 1: survey

**Questionnaire on transCervical Minimally Invasive Esophagectomy**

Since there isn’t a consensus on how to call this procedure, we open this survey with a vote on which for you could be the most appropriate name:

*MICE (Minimally Invasive transCervical oEsophagectomy)*

*TME (TransMediastinal esophagectomy)*

*MICRO (Minimally Invasive transCervical Radical Oesophagectomy)*

*SME Single-port Mediastinoscopic Esophagectomy*

*MARE (Mediastinoscopy Assisted Radical Esophagectomy)*

*MRE (Mediastinoscopic Radical Esophagectomy)*

*IMLE (Inflatable Mediastinoscopic and Laparoscopic assisted Esophagectomy)*

*MATHE (Mediastinoscope-Assisted TransHiatal Esophagectomy)*

*MELD (Mediastinoscopic Esophagectomy and Lymp node Dissection)*

*Your suggestion: _____________________________________________________________*

Section 1: General Information and learnings process

1. *How long do you perform this procedure? (In years, if less than a year answer 1)*

*_____________________________________________________________________*

1. *How many procedures have you done until now?*

*_____________________________________________________________________*

1. *In which center did you observe the procedure to learn the technique?*

_____________________________________________________________________

1. *Did you have a cadaver training before start doing the procedure on your patients?*

*yes*

*not*

*If yes, how many procedures on cadavers have you performed?* __________________

Section 2: Indications and selection of the patients

1. *Which are your indications to decide for this procedure instead of the conventional trans-thoracic esophagectomy?*

*only tumors located in the upper or middle thoracic esophagus*

*suspect lymph node metastases in the upper mediastinum*

*squamous cell carcinoma*

*previous thoracic surgery*

*history of empyema, pleuritis or others possible causes of pleural adhesions*

*all the patients*

*others*

*If others, please indicate it:* ______________________________________________

1. *Your population include:*

*patients treated with neoadjuvant chemotherapy*

*patients treated with neoadjuvant chemoradiotherapy*

*patients treated with upfront surgery (Primary surgery without neoadjuvant therapy)*

Section 3: surgical procedure

1. *How do you perform surgery:*

*Single port transcervical + laparoscopy*

*Single port transcervical + robotic abdominal phase*

*Fully robotic*

*Bilateral single port transcervical approach*

*Other*

*If other, please indicate it: _______________________________________________*

1. *Do you use the hand port during the abdominal phase?*

*yes*

*not*

1. *In which order do you perform surgery:*

*mediastinum first and then abdomen*

*abdomen first and then mediastinum*

*simultaneously*

1. *At which pressure do you set the pneumomediastinum? (mmHg) _________________*
2. *Do you use the platform in port technique to be more stable during the mediastinal dissection?*

*yes*

*not*

*Which platform do you use? (Gel point, EZ access, etc) ________________________*

1. *Which energy device do you use? __________________________________________*
2. *Which scope do you use:*

*conventional*  *5 mm*  *0°*

*flexible tip*  *10 mm*  *30°*

*45°*

1. *The patients undergo:*

*single lumen intubation*

*double lumen intubation*

1. *Do you use intraoperative neuromonitoring system to avoid injuries to the left recurrent laryngeal nerve?*

*yes, intermittent*

*yes, continuous*

*not, because I don’t think is useful*

*not, because I don’t have this technology in my hospital, but I think it could be useful*

1. *From which plane do you usually start:*

*anterior*

*posterior*

*left*

*right*

*Please explain why: ____________________________________________________*

1. *Do you usually tape the left recurrent laryngeal nerve (RLN)?*

*yes*

*not*

1. *Do you perform pyloromyotomy or pyloroplasty?*

*yes*

*not*

1. *Which route do you choose for the gastric conduit?*

*subcutaneous*

*retrosternal*

*posterior mediastinum*

1. *How do you perform the anastomosis?*

*handsewn*  *end to end*

*circular stapler*  *end to side*

*linear stapler*  *side to side*

1. *Do you position a feeding gastrostomy or jejunostomy?*

*never*

*always*

*only in selected cases*

1. *Your estimated mean operative time: (in minutes) _____________________________*
2. *Your estimated blood loss: (in milliliters) ___________________________________*
3. *Ever need for conversion to transthoracic esophagectomy?*

*yes, because the tumor infiltrated the surrounding structures*

*yes, because of hemodynamic instability caused by mediastinal inflation*

*yes, others*

*not*

*If others, please indicate it: ____________*__________________________________

1. *Which drains do you put?*

*nasogastric tube (in which POD do you remove it: ________________________)*

*cervical drain (in which POD do you remove it: __________________________)*

*mediastinal drain (in which POD do you remove it: _______________________)*

*abdominal drain (in which POD do you remove it: ________________________)*

*none*

Section 4: Post-operative course

1. *Please indicate the mean:*

- *length of ICU stays __________________________________________________*
- *length of hospital stays _______________________________________________*

1. *In which POD do you allow the oral intake? __________________________________*
2. *Prior to allow the oral intake, the patients underwent:*

*contrast esophagogram*

*upper GI endoscopy*

*CT scan*

*nothing*

Section 5: complication

1. *Your estimated percentage of anastomotic leak: ______________________________*
2. *Your estimated percentage of pulmonary complication: ________________________*
3. *Your estimated percentage of vocal cord injury/palsy: _________________________*
4. *Please indicate which type of vocal cord injury/palsy do you observed (according to the ECCG classification):*

*Type I A*  *Type II A*  *Type III A*

*Type I B*  *Type II B*  *Type III B*

1. *Do you perform laryngoscopy prior to discharge of the patient?*

*yes always*

*only in case of symptoms attributable to vocal cord injury/palsy*

1. *Did you observe other kind of complications?*

*yes*

*not*

*If yes, please indicate what kind of complications (according to the ECCG classification):* ________________________________________________________

Section 6: outcomes

1. *Which is your estimated percentage of R0 pathological resections?*

*< 50 %*

*50-60 %*

*60-70 %*

*70-80 %*

*80-90 %*

*90-100 %*

1. *Which is the mean total (mediastinal + abdominal) number of harvested lymph nodes?*

*< 15*

*15-25*

*25-35*

*> 35*

1. *Which lymph nodes stations^1^ do you systematically harvest?*

*1*

*2*

*3a*

*7*

*8a*

*8p*

*9*

*11p*

*101L*

*101R*

*104L*

*104R*

*105*

*106recL*

*106recR*

*107*

*108*

*109L*

*109R*

*110*

*111*

*112aoA*

*112pulL*

*112pulR*

References

1. Japanese classification of esophageal cancer, 11th edition: part I. Esophagus 2017; 14(1): 1–36

Supplement 2: invited centres

West China Hospital of Sichuan University, Chengdu, China

The Fifth Affiliated Hospital of Sun Yat-sen University, Guangdong, China

Jiangsu Cancer Hospital, Nanchino, China

Zhongshan Hospital of Fudan University, Shanghai, China

Mainz uniklinik, Mainz, Germany

Hiroshima City Hiroshima Citizens Hospital, Hiroshima, Japan

Dokkyo Medical University Saitama Medical Center, Koshigaya, Japan

University Hospital, Kyoto Prefectural University of Medicine, Kyoto, Japan

Oita University Hospital, Oita, Japan

The University of Tokyo Hospital, Tokyo, Japan

Keio University Hospital, Tokyo, Japan

National Cancer Center Hospital, Tokyo, Japan

Mitsui Memorial Hospital, Tokyo, Japan

Tokyo Medical and Dental University, Tokyo, Japan

University of Tsukuba Hospital, Tsukuba, Japan

Radboudumc, Nijmegen, The Netherlands

Supplement 3: Responding Centres

West China Hospital of Sichuan University, Chengdu, China

Zhongshan Hospital of Fudan University, Shanghai, China

Mainz uniklinik, Mainz, Germany

Hiroshima City Hiroshima Citizens Hospital, Hiroshima, Japan

Dokkyo Medical University Saitama Medical Center, Koshigaya, Japan

University Hospital, Kyoto Prefectural University of Medicine, Kyoto, Japan

Oita University Hospital, Oita, Japan

The University of Tokyo Hospital, Tokyo, Japan

National Cancer Center Hospital, Tokyo, Japan

Mitsui Memorial Hospital, Tokyo, Japan

University of Tsukuba Hospital, Tsukuba, Japan

Radboudumc, Nijmegen, The Netherlands
